# Mindfulness and Emotional Intelligence as Predictors of Psychological Well-Being in Athletes with Disabilities

**DOI:** 10.3390/sports13110389

**Published:** 2025-11-05

**Authors:** Diana Reklaitiene, Jolita Vveinhardt

**Affiliations:** 1 Department of Coaching Science, Lithuanian Sports University, 44221 Kaunas, Lithuania; diana.reklaitiene@lsu.lt; 2 Institute of Sport Science and Innovations, Lithuanian Sports University, 44221 Kaunas, Lithuania

**Keywords:** sport for people with disabilities, mindfulness, emotional intelligence, psychological self-feeling, psychological well-being

## Abstract

Although athletes who have disabilities face unique challenges, little is known about the interaction between mindfulness and emotional intelligence in the context of their psychological self-feeling. This study is among the first to systematically examine how mindfulness and emotional intelligence are related to stress, anxiety and depression in this population and to reveal the pathways through which the mediating effect of emotional intelligence manifests itself. A total of 95 athletes who have various types of disabilities were surveyed. The research instrument consisted of several scales: Depression, Anxiety, and Stress Scale (DASS-21), the Mindfulness Inventory for Sport (MIS), and the Emotional Intelligence Scale for Use in Sport (EIS). The data were analysed by employing descriptive statistical methods, performing correlation analysis, creating regression models, and through mediation analysis. Several significant trends were identified: professional athletes demonstrated higher mindfulness than amateurs, and higher education was associated with lower mindfulness and emotional intelligence. Mindfulness predicted better emotional competencies (B = 0.511, *p* < 0.001), which were related to lower levels of stress, anxiety, and depression (B = −0.310, *p* = 0.001), confirming a partial mediating effect (B = −0.158, 95% PI [−0.273; −0.065]). These findings add to the existing knowledge, demonstrating that in the context of sport for people with disabilities, mindfulness can improve mental health not only directly but also indirectly—through emotional intelligence. This study lays the foundation for individually tailored psychological interventions that would correspond to the specific needs of athletes who have disabilities.

## 1. Introduction

Although the media and society are gradually paying attention to sports for people with disabilities, athletes themselves are usually viewed through the medical prism of health status [1]. Significantly less attention is paid to psychosocial aspects and individual emotional experiences, which, as Silva and Howe (2022) [2] point out, have a strong impact on the athlete’s self-feeling and engagement in the social context. These aspects are particularly important, considering that physical disability is accompanied by pain, fatigue, and an increased risk of infectious diseases [3], reduced self-esteem [4], anxiety, and depressiveness [5,6].

Symptoms of stress, anxiety, and depression, understood herein as indicators of psychological self-feeling, are not only widespread among athletes with disabilities but also related to specific physical and lifestyle factors. For example, Bentzen et al. (2025) [7] found that approximately one in two members of the Swedish Paralympic team had experienced anxiety and depression over a one-year period, particularly during periods when they were suffering from injuries, illnesses, or sleep disorders. Therefore, coaches, medical specialists, and sports psychologists should pay attention not only to the physical but also to the psychological self-feeling of athletes and develop long-term psychological support strategies tailored to their experiences and risk factors [6,7].

Research conducted over the past few years shows that emotional intelligence, understood as the ability to recognize, perceive, and manage emotions [8], and mindfulness techniques, encompassing meditation, practices of accepting the present situation and oneself, can be tools that help manage stress, anxiety, and symptoms of depression in individuals of different ages and health conditions [9,10]. However, the results are not unambiguous. Many previous studies have confirmed a positive relationship between mindfulness and emotional intelligence, especially the ability to regulate emotions [11], but more recent findings suggest that this relationship may vary depending on the context and personal characteristics. For example, Guo et al. (2023) [12] note that by reducing emotional arousal, mindfulness practices can weaken the relationship with empathy and prosocial behaviour, especially among individuals with high intelligence. This suggests that the impact of mindfulness practice on emotional expression and interpersonal relationships may be contextual. Although previous research has shown that in the context of people with disabilities, emotional intelligence can act as an internal resource that helps relieve stress [13], there is still a lack of empirically grounded results demonstrating how emotional intelligence acts as an intermediate mechanism between mindfulness and psychological self-feeling indicators, such as symptoms of stress, anxiety, and depression. Greater clarity would contribute to enhancing the psychological well-being of athletes with disabilities, which includes not only the absence of psychological difficulties but also such aspects of positive self-feeling as self-esteem, life satisfaction, a sense of meaningfulness, the ability to cope with stress, maintain emotional balance, experience inner satisfaction, and constructively act in the social environment [3,4].

Although mindfulness interventions are increasingly being used to improve athletes’ psychological self-feeling, a share of research reveals more than just positive effects. Schindler et al. (2019) [14] emphasize that in certain cases, such practices can weaken moral responsibility or even encourage unethical behaviour. There have also been cases where meditation has been associated with negative phenomena: distorted self-perception, affective disorders, depressiveness, hallucinations, or false memories [15]. Such evidence suggests that the effects of mindfulness are not unambiguous and may depend on individual or contextual factors.

In the context of sport, mindfulness is most often associated with relieving psychological stress. For example, Tingaz et al. (2023) [16] found that mindfulness was negatively associated with depression, anxiety, and stress and had a positive effect on athletic performance. However, a meta-analysis performed by Kanaujia et al. (2023) in [17] showed that changes in anxiety levels were not statistically significant. Such mixed findings suggest that the effectiveness of mindfulness may depend on the nature and duration of practicing it, the sports branch, and the specific characteristics of participants. For example, de Jesus et al. (2025) [18] found that mindfulness could be beneficial for athletes with disabilities, but there were individual differences in applying this practice, and physical difficulties arose when implementing interventions. In addition, a study by Macdougall et al. (2019) [19] demonstrated that although mindfulness intervention was acceptable and safe, some effectiveness indicators (e.g., social well-being) remained unconvincing. This is particularly important when it comes to the individual needs of athletes who have disabilities, seeking more durable effects and broader applicability. Therefore, larger-scale, more tailored studies, which would take into account the specific aspects of this population, are needed.

Given the ambiguous results of previous research and the specific needs of athletes who have disabilities, this study aims to explore the links between mindfulness and emotional intelligence with stress, anxiety, and depression among athletes who have disabilities. In this context, three questions are raised: (Q1) How is mindfulness related to emotional intelligence? (Q2) What factors predict the level of mindfulness and emotional intelligence in the population of athletes with disabilities? (Q3) Does emotional intelligence act as a mediator explaining the effect of mindfulness on psychological self-feeling? All this allows not only to evaluate links between the variables under study but also to reveal possible mechanisms through which mindfulness practices or emotional intelligence contribute to the well-being of athletes who have disabilities.

## 2. Conceptual Framework

### 2.1. Mindfulness and Personal Well-Being

Mindfulness, which has become popular in the West in recent decades, is understood as a practice that allows an individual to observe, non-judgmentally accept, and reflect on personal experiences [10,20]. Mindfulness has gained popularity largely due to standardized mindfulness-based interventions that integrate the essence of traditional Eastern mindfulness practices with contemporary psychological practices to improve psychological functioning and self-feeling [20].

Three components of mindfulness-based practice include avoiding judgment of personal experiences, cultivating awareness, and focusing on the present moment [10]. That is, it enables the person to observe and reflect on his/her physical, emotional, and cognitive state without automatic judgment. The positive outcomes of this practice include reduced levels of anxiety, depression, and stress, reduced pain, and vulnerability to mental illness [11]. However, the impact of this practice on athletes is not unambiguous. The findings of a meta-analysis by Wang et al. (2023) [21] show that the level of athletic performance and awareness improved but no significant effect on athletes’ health was found.

### 2.2. The Structure of Emotional Intelligence

Emotional intelligence is defined as a set of skills that help to evaluate, express, and effectively regulate one’s own and other people’s emotions and use them for motivation, planning, and achievement [22]. In a later work, Mayer et al. (2016) [23] expand the conception of emotional intelligence as a kind of form of “hot” cognition, related to emotions and values, but distinguish it from other related constructs such as personal intelligence and social intelligence. Emotional intelligence is understood as the ability to perceive, understand, manage, and use emotions in solving problems, while personal intelligence encompasses broader self-knowledge, the ability to accurately describe one’s intrinsic world and its characteristics.

According to Olderbak et al. (2018) [24], the most commonly applied theoretical four-branch model proposed by Mayer and Salovey (1997) [25] consists of perceiving emotion, facilitating thought using emotion, understanding emotions, and managing emotions [23,24]. These branches reflect progressive skills ranging from the ability to recognize one’s own and other people’s emotions to the conscious regulation of these emotions.

### 2.3. The Mediating Role of Emotional Intelligence

Considering that mindfulness helps create emotional distance and reduces affective arousal [26,27] and that emotional intelligence enhances the perception and control of emotions [23,24], it is likely that emotional intelligence can explain how mindfulness affects psychological self-feeling. This way, emotional intelligence would act as a mediating factor, transforming the effect of mindfulness into better self-feeling.

Mediation models are often used in behavioural sciences to find mechanisms that help explain indirect relationships [28]. In this study, it is assumed that mindfulness (X) affects psychological self-feeling (Y—stress, anxiety, and depression) both directly and indirectly through emotional intelligence (M). The mediating role of emotional intelligence between mindfulness, stress, anxiety, and depression has previously been confirmed in other populations [29], but this model has not been tested in the context of sport for people with disabilities. That is, it remains unclear whether it applies to people with disabilities, whose psychological self-feeling is affected by specific factors of stress, stigma, and physical limitations. For this reason, it is worth examining whether emotional intelligence actually mediates between mindfulness and psychological self-feeling in this specific group.

## 3. Methodology

### 3.1. Participants

Since there is no unified sports register in Lithuania, it is quite difficult to determine the population of athletes who have disabilities. There is also a lack of official statistics of people who have disabilities by the type of disability (hearing, visual, mental, or physical) [30]. Some data on the population can be obtained indirectly. In 2025, the list of members and candidates for sports teams of the Lithuanian Sport Federation for the Disabled included 94 individuals, and it was also planned that in 2025, about 900 athletes would participate in various championships [31].

In total, there are three organizations uniting athletes with disabilities and conducting competitions and sports activities in Lithuania: the Lithuanian Sport Federation for the Disabled, the Lithuanian Blind Sports Federation, and the Lithuanian Deaf Sport Committee. We reviewed the websites of these organizations and the Lithuanian Paralympic Committee but did not find any recent official information about the number of participants. Thus, before conducting the study, we asked the leaders of these organizations about the adult athletes currently belonging to their organization and participating in competitions. There were about 80 athletes in the Deaf Sport Committee; 25 in the Blind Sports Federation; and about 40 athletes in the Lithuanian Sport Federation for the Disabled.

Questionnaires were prepared and distributed during two Lithuanian championships (different sports branches hold different championships). After the championships, 150 questionnaires were distributed to all athletes who had taken part in them. A total of 94 properly completed questionnaires were returned. Criteria for inclusion in the study were as follows: adult (at least 18 years old, a person able to give informed consent) participating in at least national championship level competitions, disability–blindness, deafness, or physical disability. Participants were divided into four age groups: 18–25, 26–35, 36–45, and 46–65 years old.

Assistance in completing the questionnaire was provided to deaf people, to whom questions were read and the chosen answers were marked. Everyone could choose a trusted person from their circle. The assistance did not change the content or meaning of the questions, and it is therefore considered that it did not affect the reliability of the results.

Since the population of athletes with disabilities is relatively small, the sample size is considered sufficient, taking into account several circumstances. As noted by Pensgaard and Sørensen (2002) [32], one of the main methodological challenges in research on athletes who have disabilities is the limited number of potential participants; therefore, researchers often work with samples that are close to the entire population. For example, a similar sample size was used in Martin’s (2008) [33] study, which involved 92 wheelchair basketball players, to analyse the relationship between self-efficacy and psychological self-feeling. In addition, according to Cohen’s (1992) [34] recommendations, for a multivariate regression analysis with eight independent variables, aiming for a medium effect size (f^2^ ≈ 0.15) and statistical power ≥ 0.80, approximately 92 participants are sufficient. Therefore, the sample size of this study (*n* = 95) is considered sufficient to reliably identify significant relationships even in a small population under study.

### 3.2. Measure

The survey employed three adapted psychometric scales: the Depression, Anxiety and Stress Scale (DASS) [35], the Mindfulness Inventory for Sport (MIS) [36], and the Emotional Intelligence Scale for Use in Sport (EIS) [37].

The DASS scale assessed the intensity of three components—stress, anxiety, and depression—and an overall indicator of psychological self-feeling.

The MIS scale was adapted to the context of sport and encompassed three components of mindfulness: awareness, non-judgmental, and refocusing.

The EIS scale measured five dimensions of emotional intelligence: appraisal of others’ emotions, appraisal of own emotions, regulation, social skills, and utilization of emotions.

All statements were rated on a five-point Likert-type scale, with higher scores indicating a stronger manifestation of the respective trait (except for the non-judgmental subscale, where negative values indicate an inverse relationship).

The reliability of the instrument in this study was adequate, with Cronbach’s α values ranging from 0.79 to 0.90.

### 3.3. Procedures and Ethics

The study was conducted in accordance with the ethical principles for research in social sciences, with approval from the institutional ethics committee. Participants were informed about the purpose of the study, its voluntary nature, anonymity, and their right to withdraw from the study at any time. Informed consent was obtained before filling in the questionnaire.

The questionnaires were administered in two ways: electronically and using the paper-and-pencil method. Participants were given detailed instructions on how to complete and submit questionnaires. To ensure accessibility, participants with visual or motor impairments could receive technical assistance from a trusted person chosen from their own environment (e.g., to read questions aloud or mark answers). The content and structure of the questionnaire remained the same for all participants. Therefore, this adaptation likely did not affect the reliability of the research results. All participants completed the part of the questionnaire, which collected socio-demographic data. It included questions about age, gender, type of disability, sports status (professional or amateur), sports branch, and meditation experience.

The completion took about 30–40 min, and all data were processed in accordance with the requirements of the General Data Protection Regulation, anonymized and analysed only in aggregate form. The data were processed employing descriptive statistical methods, correlations were calculated, regression models and mediations were developed.

### 3.4. Data Analysis

The collected data were analysed using descriptive statistical methods in order to determine the main characteristics of the sample. To assess the relationships between variables, Pearson correlation coefficients were calculated. The strength of the relationship was described by applying the estimates proposed by Cohen (1988) [38], where a weak relationship is *r* ≈ 0.10, a moderate relationship is *r* ≈ 0.30, and a strong relationship is *r* ≥ 0.50. Multiple regression models were created to determine the directions and strength of the effect, and mediation analysis procedures were employed to test the possible mediation effect, using the bootstrapping method to estimate confidence intervals.

## 4. Results

Of 95 respondents, 68.4% were men and 31.6 percent were women. The participants’ ages ranged from 18 to 65 years, and more than half of them (57.9 percent) were professional athletes. More than half of respondents (52.6 percent) had mobility disabilities, and the rest had sensory disabilities. Of note, 25.8 percent held a university degree, 29 percent held a vocational degree, and the rest studied at school, colleges, and universities. Approximately one in three athletes has been doing sports for more than twenty-five years, which indicates their significant experience and involvement in sports. Such diversity reflects different sociodemographic groups and experiences as well as allows for a comprehensive examination of links between mindfulness, emotional intelligence, and psychological self-feeling (stress, anxiety, and depression) in the context of sport for people with disabilities.

### Relationships Between Emotional Intelligence, Mindfulness, and Psychological Self-Feeling

First, a correlation analysis of emotional intelligence and mindfulness (including overall scores) was performed; its results are presented in Table 1.

Thus, the results show that mindfulness is positively correlated with most components of emotional intelligence, especially with the regulation of emotions (r = 0.452, *p* < 0.01) and the overall estimate (r = 0.522, *p* < 0.01). Meanwhile, the non-judgmental component showed weak negative relationships with several aspects of emotional intelligence, and the relationship with social skills was statistically insignificant (r = −0.18, *p* = 0.09). As non-judgmental scores are increasing, the estimates of emotional intelligence components are significantly decreasing, including estimates of appraisal of other people’s emotions, regulation of emotions, and overall emotional intelligence (r = −0.30, *p* < 0.01). This suggests that a higher level of non-judgmental self-acceptance is related to lower indicators of the ability to recognize, regulate, and purposefully utilize emotions.

When examining the relationships between emotional intelligence and psychological self-feeling (stress, anxiety, and depression), it was found that only appraisal of others’ emotions was statistically significantly correlated with all indicators reflecting negative psychological self-feeling. The strongest relationship was found with the overall estimate of self-feeling (r = 0.293, *p* = 0.004) and stress (r = 0.304, *p* = 0.003). Meanwhile, a weaker but significant correlation was identified with anxiety (r = 0.228, *p* = 0.026) and signs of depression symptoms (r = 0.243, *p* = 0.018). However, only two of the mindfulness components (non-judgmental and overall scale estimate) showed statistically significant negative relationships with psychological self-feeling (Table 2).

The non-judgmental component was strongly and consistently correlated with all four indicators of negative psychological self-feeling (r fluctuated from −0.336 to −0.466, *p* < 0.001), indicating that the better the athletes were able to accept their experiences without judgment, the lower the levels of their experienced stress, anxiety, and depression. Links of awareness and refocusing components with self-feeling of athletes with disabilities were not significant.

Factors predicting the level of mindfulness among athletes with disabilities were revealed by the results of regression analysis. Independent variables included sociodemographic indicators (age, gender, and education), peculiarities of doing sports (duration, level, and sports group), experience in practicing meditation, the type of disability, and the indicator showing psychological self-feeling (composed of the sum of stress, anxiety, and depression estimates).

The results show that the regression model is statistically significant (F(10, 76) = 3.01, *p* = 0.003) and explains 24.9% (R^2^ = 0.249) of the variance in the mindfulness estimate (Table 3).

Psychological self-feeling (stress, anxiety, and depression) was the most significant predictor of mindfulness (β = −0.423, *p* < 0.001), meaning that the higher the levels of anxiety, depression, and stress, the lower the level of mindfulness. The level of doing sports (professional vs. amateur) was significantly associated with a higher estimate of mindfulness (β = 0.318, *p* = 0.011). That is, professional athletes distinguish themselves by a higher level of mindfulness than amateurs. Meanwhile, education had a negative effect on mindfulness (β = −0.256, *p* = 0.016). This suggests that mindfulness may be weaker in athletes who have disabilities and higher education. Gender, age, duration of exercising, meditation experience, and the type of disability were not statistically significant predictors (*p* > 0.05).

In order to determine which factors predict mindfulness estimates, including psychological self-feeling components (stress, anxiety, and depression) as separate predictors, the second regression model was built (Table 4).

The results of regression analysis show that the depression signs indicator has a tendency to approach a statistically significant effect on mindfulness (β = −0.270, *p* = 0.063). The anxiety and stress estimates also did not have a statistically significant effect (*p* > 0.1), and their standardized coefficients were insignificant.

To examine which factors predict the overall emotional intelligence indicator among athletes who have disabilities, a regression model was constructed with independent variables, which encompassed demographic indicators, athletic factors, and psychological self-feeling components (stress, anxiety, and depression). The model was statistically significant (F(10, 94) = 3.692, *p* < 0.001) and explained 28.2% (R^2^ = 0.282) of the overall variance in emotional intelligence (Table 5).

Emotional intelligence was statistically significantly predicted by stress level (β = 0.463, *p* = 0.004) and education (β = −0.303, *p* = 0.007), indicating that a higher level of education was associated with lower emotional intelligence scores. A statistically significant negative effect of meditation on emotional intelligence estimates was also found (β = −0.199, *p* = 0.043). The impact of other factors (anxiety, depression, duration of exercising, gender, age group, type of sports, sports group, and type of disability) was not statistically significant (*p* > 0.05).

**Figure 1:**
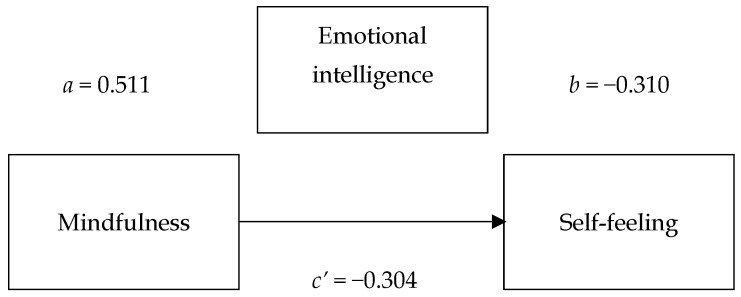
The mediation model.

To answer Q3, mediation analysis was performed. It was found that emotional intelligence acted as a partial, statistically significant mediator, explaining the effect of mindfulness on negative psychological self-feeling (stress, anxiety, depression). Figure 1 presents a model of mediation pathways. A more detailed review of the statistical results is provided in Table 6.

The results show that mindfulness significantly predicted emotional intelligence indicators (path a, B = 0.511, *p* < 0.001), while emotional intelligence significantly affected the negative self-feeling estimate (path b, B = −0.310, *p* = 0.001). There was also a statistically significant and direct effect of mindfulness on the overall self-feeling indicator (path c’, B = −0.304, *p* = 0.003), but it was weaker than the overall effect (path c, B = −0.462, *p* < 0.001). The indirect effect through emotional intelligence was statistically significant, and the 95% confidence interval did not include zero (indirect effect B = −0.158, 95% CI [−0.273, −0.065]).

## 5. Discussion

This study aimed to investigate links of mindfulness with stress, anxiety, and depression in the context of athletes who had disabilities and to reveal mechanisms through which mindfulness practices and emotional intelligence contribute to athletes’ psychological well-being. Since the interrelationships between these constructs in the population of people who have disabilities have been poorly studied to date, questions were consistently raised that helped reveal not only direct but also indirect links between mindfulness and indicators of psychological self-feeling. The answers to these questions create prerequisites for a deeper understanding of the theoretical interrelationships between these phenomena and provide the basis for practical interventions aimed at strengthening the psychological well-being of athletes who have disabilities.

The first research question (Q1) aimed to find out how mindfulness was related to emotional intelligence, since it is namely this connection that creates conditions for further analysis and allows us to understand how mindfulness abilities can be linked to appraisal, regulation, and utilization of emotions in the context of sport for people with disabilities. It was found that mindfulness was significantly correlated with most components of emotional intelligence (except social skills). Social skills require the ability to recognize and understand the emotions of others, while Nadler et al. (2020) [39] found that mindfulness intervention participants had reported improvements in all components of emotional intelligence except empathy. Considering that social skills are closely related to the ability of empathy, no increase in this component might have determined a weaker relationship between mindfulness and social skills. This is consistent with previous observations that mindfulness is more likely to enhance intrapersonal emotional intelligence (self-knowledge and regulation of emotions) than interpersonal emotional intelligence (empathy, social communication) [39,40]. In addition, according to the results of a meta-analysis performed by Li et al. (2023) [41], mindfulness was associated with an increase in intrinsic motivation and with meeting basic psychological needs, such as autonomy, competence, and relatedness. This interaction suggests that mindfulness practices primarily promote intrinsic regulation and self-acceptance that is focused on the person’s internal experience rather than on the demonstration of external social skills. This may explain why mindfulness was not correlated with the “social skills” scale in this study.

Notably, a higher level of non-judgmental self-acceptance was associated with lower scores of the ability to recognize, regulate, and use emotions appropriately. This result may seem contradictory, but it is consistent with the mechanism identified by Josefsson et al. (2017) [42], according to which a higher level of mindfulness (non-judgmental attitude) can reduce the tendency to rumination and enhance the ability of “reperceiving” emotional experiences. This allows athletes to “cool down” intense emotions more quickly, limit their duration, and reduce the need to actively apply conscious emotion recognition or regulation strategies. As a result, emotional intelligence indicators measured by the questionnaire may be lower, not due to poorer skills but due to a lower need to apply them.

On the other hand, the non-judgmental component was found to be strongly and consistently correlated with stress, anxiety, and depression, indicating that the better athletes are able to accept their experiences without judgment, the lower their level of psychological self-feeling. This result coincides with previous research showing that the non-judgmental acceptance approach can mitigate stressors experienced by athletes and support their psychological well-being [7,43]. According to Gu et al. (2015) [44], the said component helps reduce the automatic appraisal of negative thoughts, leading to reduced affective arousal and maintenance of better emotional balance, which can explain strong and consistent correlations with all three psychological state indicators.

Examining the role of mindfulness and emotional intelligence in predicting psychological self-feeling (Q2), it turned out that psychological self-feeling (levels of stress, anxiety, and depression) was the strongest predictor of mindfulness, revealing a negative association: higher indicators were related to lower levels of mindfulness. In addition, it was found that the level of doing sports (professional vs. amateur) significantly predicted mindfulness. Professional athletes distinguished themselves by higher mindfulness than amateurs. This can be related to higher requirements for psychological preparation and skill development, including concentration of attention and regulation of emotions. It should be emphasized that education had a negative effect on mindfulness, which could indicate that athletes with higher education may rely more on cognitive rather than mindfulness-based decision-making processes. For example, Scharfen and Memmert (2019) [45] argue that professional athletes are more characterized by developed cognitive abilities that are of great importance for decision-making. Furthermore, Waterworth et al. (2020) [46] highlight that higher education may promote a more analytical, fact-based, and logical decision-making style. This may indicate that athletes with disabilities and higher education tend to evaluate situations primarily cognitively.

These interpretations are indirectly supported by the findings of a study conducted by Noone and Hogan (2017) [47]. The results show that individuals with stronger cognitive approaches, such as a high need to seek knowledge or active open-minded thinking, benefited less from mindfulness interventions. Although their study did not directly examine the role of education, the observed interaction between cognitive style and mindfulness may help explain the negative link found in this study. It is possible that individuals with higher education are more actively engaged in cognitive processes that are less compatible with experiential, non-judgmental aspects of mindfulness. Liu and Nesbit (2024) [48] revealed in their meta-analysis that a higher need for cognition was associated with higher academic achievement, since such individuals were more inclined to deep analysis of information, reflection, and coping with cognitively complex tasks. All this suggests that the abundance of cognitive resources or the tendency towards cognitive analysis, which are usually characteristic of persons with higher-level education, may reduce their sensitivity to mindfulness practices that emphasize non-analytical, open, and non-judgmental attentiveness.

Meanwhile, demographic characteristics, meditation experience, and the type of disability did not have a significant effect, suggesting that these factors were not essential predictors of mindfulness in this context.

Emotional intelligence was significantly predicted by stress (β = 0.463, *p* = 0.004), education (β = −0.303, *p* = 0.007), and meditation practice (β = −0.199, *p* = 0.043). Fiorilli et al. (2021) [49] found that when experiencing stress, athletes who had disabilities actively used various coping mechanisms related to emotional reactions. That is, having encountered stressful situations, athletes can better mobilize and improve their ability to recognize and regulate emotions in order to effectively overcome challenges. The negative effect of education might have been related to cognitive orientation and less attention to emotional aspects [46], while the impact of meditation confirms previous research demonstrating that meditation does not always have a positive effect on emotional intelligence. For example, the study of Valim et al. (2019) [50] showed that when applying mindfulness meditation, no positive effect on the regulation of emotions was observed. Furthermore, decreasing emotional arousal may result in weakening of empathy [39,44], which is important for perceiving other people’s emotions and social skills. The role of meditation intention, which is oriented to athletic achievements, cannot be ruled out either.

Being related to the person’s ability to perceive and regulate emotions, emotional intelligence also plays an important role in moral decision-making. As pointed out by Vveinhardt and Deikus (2025) [51], moral decision-making is considerably affected by emotions and intuition, and synderesis allows one to correct mistakes made in this area. This indicates a broader impact of emotional intelligence, which, in addition to reducing stress and anxiety, can also promote more conscious and ethical behaviour.

Finally, the results of mediation analysis (Q3) showed that in the sample of athletes with disabilities, emotional intelligence acted as a partial mediator between mindfulness and negative psychological self-feeling (stress, anxiety, and depression). Higher level of mindfulness was associated with a better ability to perceive, regulate, and purposefully use emotions, and this ability was related to lower indicators of psychological self-feeling. The direct effect of mindfulness on self-feeling remained significant even after inclusion of emotional intelligence in the model, although its strength reduced compared with the overall effect. This shows that emotional intelligence conveys some, but not all, of the effects of mindfulness on psychological well-being. A previous study in an adolescent population demonstrated a mediating role of emotional intelligence between mindfulness and both anxiety and depression [29]. In another study of the adult population, Iau et al. (2025) [52] concluded that the ability to understand and use one’s emotions might be one of the possible mechanisms through which mindfulness reduced anxiety, depression, and contributed to well-being.

The results of this study can be useful in developing the theoretical discussion on mindfulness, emotional intelligence, depression, anxiety, and stress in sports psychology, especially in the context of athletes who have disabilities. Previous studies have mainly focused on the examples of healthy athletes or the general population, while the findings of this study reveal that emotional intelligence partially explains the impact of mindfulness on psychological self-feeling among athletes who have disabilities too. Furthermore, the unusual relationship between the non-judgmental component and emotional intelligence indicators, identified in this study, provides a basis for further developing the discussion about the effects of different dimensions of mindfulness on individual components of emotional intelligence.

In practice, the findings can be valuable to sports psychologists, coaches, and rehabilitation specialists, who assist athletes with disabilities. Mindfulness and emotional intelligence practices integrated into training programs can not only directly reduce stress and anxiety but also indirectly improve self-feeling through emotion recognition and regulation abilities. Such a dual mechanism of impact can be particularly important for the long-term well-being of athletes who have disabilities. In this context, it is recommended that specialists take into account certain individual differences that determine the outcome of practices.

Considering the results of this study, it is recommended that sports psychologists and coaches working with athletes who have disabilities should incorporate structured emotional intelligence and mindfulness development programs. One such framework was presented by Laborde et al. (2022) [53], who proposed practical interventions aimed at the development of five emotional competencies: identification, expression, understanding, regulation, and use of emotions. These aspects can be developed through activities adapted to sport, such as ‘discussion of emotions’, ‘memory of emotions’, and ‘sports awareness’, which are based on the tripartite model of emotional intelligence (of knowledge, abilities, and traits). This framework can be combined with the MAC (Mindfulness–Acceptance–Commitment) protocol (Gardner & Moore, 2007) [54], which integrates present-moment awareness, acceptance of internal experiences, and value-based actions. Such preliminarily proposed protocols could be further adapted to the sports environment, taking into account the specific needs and context of athletes who have disabilities.

The study has several limitations. First, the cross-sectional design does not allow to make firm conclusions about causal relationships; therefore, a longitudinal study would be needed in the future. Second, although the sample size is sufficient for statistical analysis, it is still limited, and the participants were from a single country, which limits the possibilities of making broader generalizations. Third, we were unable to find other studies that simultaneously examined the same concepts (mindfulness, emotional intelligence, depression, anxiety, and stress) in the context of sport for people with disabilities. For this reason, it was not possible to directly compare the obtained results with previous studies. Fourth, self-assessment questionnaires can be affected by social desirability and subjective perception. However, in order to mitigate these risks, several methodological solutions were applied: validated and reliable scales (DASS, MIS, and EIS) were used, and the data were collected by selecting both electronic and paper-and-pencil questionnaires adapted for participants with different types of disabilities.

Finally, the study was conducted in Lithuania, whose sociocultural context might have influenced the interpretation and application of findings in other cultures. In Lithuania, there is a lower tendency to openly express emotions, compared with, for example, the English-speaking culture (Šeškauskienė, 2020) [55], and mindfulness practices are not yet widespread; therefore, the experiences of research participants in these areas may be limited. Social stigma associated with disability (Grigaitė et al., 2025) [56] may also have influenced the openness of responses. To manage this limitation, the participants’ anonymity and the possibility of choosing a response method that was acceptable to them were ensured. Although due to cultural peculiarities, the results of the study may not be fully transferable to other contexts, the applied ethical and methodological measures helped to reduce the risk of cultural influence.

## 6. Conclusions

This is probably one of the first studies that systematically examines links between mindfulness, emotional intelligence, and psychological self-feeling components (stress, anxiety, and depression) in the context of sport for people with disabilities. The findings have confirmed the partial mediating effect of emotional intelligence, indicating that the abilities to appraise, regulate, and utilize emotions can be one of the important mechanisms through which mindfulness practices influence the psychological well-being of people who have disabilities. This not only expands theoretical knowledge about the interaction between psychological constructs in sport for people with disabilities but also provides new insights that can contribute to the development of intervention mechanisms to improve psychological self-feeling. In this context, it is important to note that in assessing psychological self-feeling, generalized indicators of stress, anxiety, and depression are more useful than individual symptoms. The obtained results suggest the potential for applying complex interventions that would include both mindfulness and emotional intelligence enhancement methods.

Considering the limitations of this study, it is recommended that future research should use a longitudinal or intervention-based design that would allow for assessment of causal relationships between mindfulness, emotional intelligence, and psychological self-feeling indicators. Longitudinal studies would provide a better understanding of how consistent mindfulness practice affects emotional management skills over time, while interventional studies would allow us to evaluate the effectiveness of specific programs (e.g., mindfulness training sessions adapted for sports) in a group of athletes who have disabilities. It would also be appropriate to investigate the impact of different mindfulness components (e.g., attentiveness, non-judgment, and reorientation) on individual dimensions of emotional intelligence. Given the observed role of education, future research could examine in more detail the interaction between cognitive styles and the effectiveness of mindfulness practices. Finally, larger and more culturally diverse samples could help evaluate the extent to which the research findings are universal and applicable to other cultural settings.

## Figures and Tables

**Table 1:** Relationships of emotional intelligence with mindfulness variables.

	Appraisal of Others’ Emotions	Appraisal of Own Emotions	Regulation	Social Skills	Utilization of Emotions	Emotional Intelligence (Overall)
Awareness	0.461 **	0.572 **	0.601 **	0.381 **	0.354 **	0.596 **
Non-judgmental	−0.304 **	−0.261 *	−0.346 **	−0.175 (ns)	−0.120 (ns)	−0.302 **
Refocusing	0.371 **	0.498 **	0.513 **	0.361 **	0.564 **	0.600 **
Mindfulness (overall)	0.311 **	0.477 **	0.452 **	0.333 **	0.457 **	0.522 **

Notes: *p* < 0.05 (*); *p* < 0.01 (**); ns—non-significant (*p* > 0.05).

**Table 2:** Relationships of mindfulness components with negative self-feeling in athletes.

	Stress	Anxiety	Depression	Overall
Awareness	0.055	0.128	−0.060	0.042
Non-judgmental	−0.466 **	−0.384 **	−0.336 **	−0.447 **
Refocusing	−0.009	−0.172	−0.132	−0.117
Overall	−0.258 *	−0.245 *	−0.318 **	−0.312 **

Notes: *p* < 0.05 (*); *p* < 0.01 (**).

**Table 3:** Prognostic model of mindfulness. R^2^ = 0.249.

Coefficients ^a^						
Model		Unstandardized Coefficients		Standardized Coefficients	t	Sig.
		B	Std. Error	Beta		
1	(Constant)	57.211	5.525		10.355	<0.001
	Stress, anxiety, and depression	−0.246	0.061	−0.423	−4.040	<0.001
	Practice meditation	−1.704	1.422	−0.110	−1.198	0.234
	Type of disability	−0.167	1.276	−0.016	−0.131	0.896
	Years of doing sports	0.013	0.347	0.004	0.036	0.971
	Does sports as a professional or as an amateur	4.662	1.785	0.318	2.611	0.011
	Sports group	1.742	0.732	0.235	2.378	0.020
	Gender	−1.076	1.504	−0.069	−0.715	0.477
	Age group	0.071	0.870	0.010	0.082	0.935
	Education	−1.163	0.472	−0.256	−2.463	0.016

^a^ Dependent Variable: Mindfulness.

**Table 4:** Prognostic model of mindfulness by detailing psychological self-feeling. R^2^ = 0.241.

Coefficients ^a^						
Model		Unstandardized Coefficients		Standardized Coefficients	t	Sig.
		B	Std. Error	Beta		
1	(Constant)	56.771	5.569		10.193	<0.001
	Practice meditation	−1.618	1.443	−0.105	−1.122	0.265
	Type of disability	−0.007	1.290	−0.001	−0.005	0.996
	Years of doing sports	0.004	0.350	0.001	0.010	0.992
	Does sports as a professional or as an amateur	4.907	1.808	0.335	2.714	0.008
	Sports group	1.539	0.760	0.208	2.026	0.046
	Gender	−1.372	1.543	−0.088	−0.889	0.376
	Age group	0.002	0.899	0.000	0.002	0.998
	Education	−1.215	0.480	−0.268	−2.529	0.013
	Stress	−0.009	0.230	−0.006	−0.040	0.968
	Anxiety	−0.347	0.236	−0.210	−1.472	0.145
	Depression	−0.392	0.207	−0.270	−1.887	0.063

^a^ Dependent Variable: Mindfulness.

**Table 5:** Prognostic model of emotional intelligence.

Coefficients ^a^						
Model		Unstandardized Coefficients		Standardized Coefficients	t	Sig.
		B	Std. Error	Beta		
1	(Constant)	79.256	10.642		7.447	<0.001
	Practice meditation	−5.670	2.757	−0.199	−2.056	0.043
	Type of disability	2.223	2.465	0.119	0.902	0.370
	Years of doing sports	−0.288	0.670	−0.055	−0.431	0.668
	Does sports as a professional or as an amateur	3.121	3.455	0.115	0.903	0.369
	Sports group	1.418	1.452	0.104	0.977	0.332
	Gender	−3.650	2.947	−0.127	−1.238	0.219
	Age group	0.095	1.717	0.008	0.056	0.956
	Education	−2.537	0.918	−0.303	−2.763	0.007
	Stress	1.313	0.440	0.463	2.986	0.004
	Anxiety	−0.546	0.451	−0.179	−1.212	0.229
	Depression	−0.711	0.396	−0.266	−1.793	0.077

^a^ Dependent variable: Emotional intelligence.

**Table 6:** Results of mediation analysis.

Path	B (Unstandardized Coefficient)	SE	t	*p*-Value	95% PI (Bootstrap)
a: mindfulness → emotional intelligence	0.511	0.074	6.89	<0.001	[0.365, 0.655]
b: emotional intelligence → self-feeling (stress, anxiety, depression)	−0.310	0.094	−3.30	0.001	[−0.495, −0.124]
c: mindfulness → self-feeling (overall impact)	−0.462	0.095	−4.87	<0.001	[−0.648, −0.276]
c’: mindfulness → self-feeling (direct effect)	−0.304	0.098	−3.10	0.003	[−0.496, −0.112]
indirect effect (a × b)	−0.158	–	–	–	[−0.273, −0.065]

## Data Availability

The raw data supporting the conclusions of this article will be made available by the authors upon request.
